# Prophylactic effects of alpha-blockers, Tamsulosin and Alfuzosin, on postoperative urinary retention in male patients undergoing urologic surgery under spinal anaesthesia

**DOI:** 10.1590/S1677-5538.IBJU.2015.0256

**Published:** 2016

**Authors:** Ali Akkoc, Cemil Aydin, Ramazan Topaktas, Mahir Kartalmis, Selcuk Altin, Kenan Isen, Ahmet Metin

**Affiliations:** 1 Department of Urology, Gazi Yasargil Training and Research Hospital, Diyarbakir, Turkey;; 2 Department of Urology, Selahaddin Eyyubi State Hospital, Diyarbakir, Turkey;; 3 Department of Urology, Faculty of Medicine, Abant Izzet Baysal University, Bolu, Turkey

**Keywords:** Adrenergic alpha-Antagonists, Postoperative Period, Urinary Retention, Anesthesia, Spinal

## Abstract

**Purpose:**

Postoperative urinary retention (POUR) is one of the most common complications after surgical procedures under spinal anaesthesia. Recent studies have shown the beneficial effects of alpha-adrenergic blockers in preventing POUR. The aim of this prospective study was to investigate and compare the prophylactic effects of tamsulosin and alfuzosin on POUR after urologic surgical procedures under spinal anaesthesia.

**Materials and Methods:**

A total of 180 males who underwent elective urologic surgery were included in this study. The patients were randomly allocated into three Groups. The Group I received placebo. Patients in Group II were given 0.4mg of tamsulosin orally 14 and 2 hours before surgery. Patients in Group III were given 10mg of alfuzosin ER orally 10 and 2 hours before surgery. All patients were closely followed for 24 hours postoperatively and their episodes of urinary retentions were recorded.

**Results:**

There were 60 patients in each Group. Their mean age was 35.95±15.16 years. Fifteen patients in Group I (25%), 3 patients in Group II (5%) and 4 patients in Group III (6.7%) required catheterization because of urinary retention. In tamsulosin group and alfuzosin group, there were a significantly lower proportion of patients with POUR compared with the placebo Group (p=0.002 and p=0.006). The beneficial effects of tamsulosin and alfuzosin on POUR were similar between both Groups (p=0.697).

**Conclusion:**

This study suggests that the use of prophylactic tamsulosin or alfuzosin can reduce the incidence of urinary retention and the need for catheterization after urologic surgical procedures under spinal anaesthesia.

## INTRODUCTION

Spinal anaesthesia is a common regional anaesthesia technique performed by anaesthesiologists since 1898 (1). It has some complications such as hypotension, bradycardia, cardiac arrest, nausea-vomiting, transient neurologic problems, headache, pruritus and urinary retention (2).

Postoperative urinary retention (POUR) has generally been defined as the inability to pass any urine in the presence of a percussible or palpable bladder after surgery, but the definition varies widely. POUR is common and represents between 5% to 70% of all surgeries (3). It occurs more frequently in lower urinary tract, perineal, inguinal, orthopaedic, gynecologic, and anorectal surgeries after spinal anaesthesia.

Urethral catheterization, a mainstay of initial management for patients with POUR, is associated with some complications and increases in cost of care (3, 4). Both the health and financial costs of retention are considerable, because it can cause urinary tract infections and necessitate catheterization, which can in turn result in urethral strictures, prolonged hospital stays, and additional operations. Therefore, pharmacological therapy is viewed as an interesting option for patients developing urinary retention following surgery.

Urinary retention in the postoperative period has two main causes. The first is mechanical obstruction of lower urinary tract and the second is altered neural control of the bladder and detrusor mechanism, most commonly due to analgesic drugs (5). Additionally, high sympathetic activity increases the risk of urinary retention. Therefore, inhibition of alpha-adrenergic receptors located on the bladder neck and proximal urethra may prevent POUR (3). Tamsulosin and alfuzosin are safe selective alpha1-adrenergic receptor blockers characterized by their favorable side effect profiles (6, 7). There is currently little published data on the incidence and treatment of urinary retention after spinal anaesthesia in urologic surgery procedures. We think that prophylactic effects of alpha-blockers on POUR after urologic surgical procedures under spinal anaesthesia have not been investigated adequately.

The aim of the present study was to investigate the prophylactic effects of tamsulosin and alfuzosin on the prevention of urinary retention in male patients after spinal anaesthesia in urologic surgery procedures.

## MATERIALS AND METHODS

From January 2010 through October 2014, a total of 180 male patients aged 18 to 69 years who underwent elective inguinal, penile, scrotal and perineal surgery under spinal anesthesia were included in this study. The study was performed in accordance with the Declaration of Helsinki and approved by the local ethics committee of Diyarbakir Training and Research Hospital. All patients provided informed consent.

The exclusion criteria were patients who had severe lower urinary tract symptoms before surgery (according to AUA-American Urological Association-symptom score), active urinary tract infection, medications that could affect voiding function such as alpha-agonists/antagonists and cholinergic/anti-cholinergic drugs, urinary incontinency, previous history of lower urinary tract surgery and history of neurological, urological or systemic disease (such as multiple sclerosis, prostate cancer, diabetes mellitus).

The patients were submitted to physical examination, blood analysis, electrocardiogram, chest X-ray, urinalysis, uroflowmetry and ultrasonographic investigation (measurement of prostatic volume and postvoid residual urine volume). The patients were randomly allocated into three Groups. In Group I (placebo), the patients were given two doses of placebo orally 2 and 12 hours before surgery. The patients in Group II (Tamsulosin) were given 0.4mg of tamsulosin orally 14 and 2 hours before surgery. The patients in Group III (Alfuzosin) were given 10mg of alfuzosin ER (extended release) orally 10 and 2 hours before surgery. The whole patients voided before transfer to the operating area. Ringer’s lactate solution was infused at a rate of 10mL/kg/h during surgery and 30mL/kg/24h after operation. Surgery was performed under spinal anesthesia using 14-20mg bupivacaine. The patients were followed for 24 hours postoperatively. Nonsteroidal anti-inflammatory drugs were ordered for postoperative analgesia. Opioid analgesics were not applied to any patient in the postoperative period. The diagnosis of POUR was proved when the patient had a painful and palpable mass in suprapubic area, and was unable to void during the first 12 hours after surgery. It was confirmed by emptying of more than 500mL of urine by catheterization. A 14-French Foley catheter was placed to decompress the bladder of patients who could not urinate after surgery. Operation times, patient’s age, urinary symptom scores of patients and urinary retentions were recorded and parameters were compared among three Groups.

All statistical evaluations were performed by the Statistical Package for Social Sciences (SPSS) software for Windows, version 15.0 (SPSS Inc., Chicago, IL, USA). Statistical analysis was accomplished by use of ANOVA (Analysis of variance), chi-square and Mann-Whitney U tests with a p-value of less than 0.05 considered significant.

## RESULTS

A total of 180 patients who were assigned to placebo Group (Group I, n=60), tamsulosine Group (Group II, n=60) and alfuzosin Group (Group III, n=60) were included in the analysis. Inguinal surgery (especially varicocelectomy, n=89) was the most frequent surgery in all Groups. The other types of surgical procedures were hydrocelectomy (n=29), spermatocelectomy (n=8), epididymal cyst excision (n=6), scrotal orchiectomy (n=14), inguinal orchiectomy (n=7), orchiopexy (n=8), peyronie’s disease and congenital penile curvature surgery (n=15), perineal ectopic testis surgery (n=1), perineal mass surgery (n=1), lymphangioma circumscriptum surgery (inguinal and perineal, n=2). The mean age of patients was 35.95±15.16. No statistically significant differences were found among three Groups in terms of age (p=0.819), duration of surgery (p=0.10) and severity of preoperative urinary symptom scores (p=0.995). In Group one, 15 patients required catheterization with a mean urine volume of 670mL at catheterization. In Group two, 3 patients required catheterization with a 650mL mean urine volume. In Group three, 4 patients required catheterization with a 720mL mean urine volume. Thus, 25% of patients in Group I, 5% of patients in Group II and 6.7% of patients in Group III had urinary retention. In tamsulosin Group, there was a significantly lower proportion of patients with POUR compared with the placebo Group (p=0.002). In alfuzosin Group, there was a significantly lower proportion of patients with POUR compared with the placebo Group, too (p=0.006). The beneficial effects of tamsulosin and alfuzosin on POUR were similar in both Groups (p=0.697) (Table-1). Two patients in tamsulosin Group and one patient in alfuzosin Group showed some side effects at 24 hours follow-up. All three patient’s experienced vomiting and dizziness. Side effects were mild and did not lead to exclusion of patients from the study.

**Table 1:** Clinical features and demographic characteristics of patients in three groups and comparison of all groups in term of POUR.

	Group I (Placebo)	Group II (Tamsulosin)	Group III (Alfuzosin)	ANOVA F Test (F) or chi-square test (X^2^)	p value
Number of patients	60	60	60		
Mean age±SD (year)	34.95±15.21 (18-67)	36.30±15.22 (18-69)	36.60±15.26 (18-68)	F=0.200	0.819
**Pre-operative urinary**				X^2^=0.196	0.995
Symptoms*	37 (61.6%)	38 (63.3%)	39 (65%)		
No	16 (26.7%)	15 ( 25%)	15 (25%)		
Mild Moderate	7 (11.7%)	7 (11.7%)	6 (10%)		
**Region of surgery**					
Inguinal	34 (56.7%)	36 (60%)	34 (56.7%)		
Penile	6 (10%)	4 (6.7%)	5 (8.3%)		
Scrotal	18 (30%)	19 (31.6%)	20 (33.3%)		
Perineal	2 (3.3%)	1 (1.7%)	1 (1.7%)		
Mean operation time±SD (minute)	48.58±12.69 (27-78)	52.28±13.34 (29-85)	53.63±13.72 (28-84)	F=2.333	0.100
Number of patients with POUR	15(25%)	3(5%)	4(6.7%)		
Comparison of Group I and Group II in term of POUR				X^2^=9.412	0.002
Comparison of Group I and Group III in term of POUR				X^2^=7.566	0.006
Comparison of Group II and Group III in term of POUR				X^2^=1.52	0.694

*According to AUA (American Urological Association) symptom score.

There was no statistically significant difference in age, IPSS (International Prostate Symptom Score) and operation time between patients who developed urinary retention and those who did not (Table-2).

**Table 2:** Demographic data and clinical features of the all patients who developed POUR and those who did not.

	POUR (+) (n=22)	POUR (-) (n=158)	Z Score	p Value
Mean Age±SD (years)	38.86±14.558	35.54±15.243	-1.137	0.256
Mean IPSS±SD	3.23±3.161	2.80±4.638	-1.499	0.134
Mean Operation Time±SD (minute)	49.23±11.467	51.82±13±597	-0.798	0.425

## DISCUSSION

POUR is a common complication after spinal anaesthesia in urologic and other surgical procedures. It is a medical emergency requiring prompt action. The incidence of urinary retention after spinal anaesthesia ranges from 0% to 69% (8). The data on regional anesthesia and its effect on POUR is more consistent in other fields. Spinal anesthesia has been shown to increase rates of urinary retention in orthopaedic, podiatric, and hernia surgery (9). POUR causes pain and discomfort after surgery and catheterization for resolving it, may lead to urethral injury or stricture or urinary tract infection and increase cost and work load and hospitalization period (10).

Three methods have been used to diagnose POUR: History and physical examination, ultrasonographic imaging of bladder and bladder catheterization (11). We used two practical methods for diagnose of POUR: 1-History and physical examination (lower abdominal pain and discomfort and palpation or percussion of bladder in suprapubic area); 2-Bladder catheterization. Many studies indicate that urine retention can be diagnosed when the patient cannot urinate at bladder volumes above 400–600mL (12, 13). The average urine volumes were above 500mL in all of our patients with POUR. We think that diagnosis of POUR by history and physical examination instead of ultrasonography was one of the limitation of this study.

Disturbances of micturition are common in the first 24 hours after spinal anesthesia. There is a higher frequency of these disturbances after bupivacaine than lidocaine spinal anesthesia (2, 14). After administration of spinal anesthesia with bupivacaine or tetracaine, the micturition reflex is very rapidly eliminated. Detrusor muscle contraction is restored to normal 7-8 hours after the spinal injection. On average, patients recover enough motor function to be mobilized 1–2 hours before the micturition reflex returns (2). Kamphius et al. found that motor blockade following bupivicaine spinals lasted 148±76 minutes compared to detrusor blockade of 462±61 minutes (15).

Many factors contribute to the development of POUR. These include history of underlying disease, the direct effects of anesthetic agents on the bladder, excessive perioperative fluid intake, traumatic instrumentation, pelvic dissection, diminished awareness of bladder sensation, increased outlet resistance, immobilization after the surgery, postoperative pain and use of narcotics, type of anesthesia, duration of surgery, gender and age (3, 11). The stress response to surgery and especially postoperative pain increase sympathetic tone. When ephinephrine is injected intraperitoneally in rats, the intravesical pressure increases without raising urine output, suggesting that ephinephrine increases internal urethral sphincter tone by acting on alpha receptors in the bladder neck (16). So, the sympathetic stimulation influence the relaxation of the detrusor and close the internal urethral sphincter. The resultant stimulation of the alpha receptors in the internal urethral sphincter leads to increased pressure on the bladder neck and potentially to POUR (3). Micturition reflex might be inhibited by the high sympathetic activity after surgery. Alpha-blocker premedication might have inhibitory effect on the elevated sympathetic activity and therefore, prevent acute urinary retention after surgery.

Petros and colleagues reviewed 295 inguinal herniorrhaphies in men. They found use of spinal anesthesia, age less than 53 years, and perioperative fluid less than 1200mL all significantly reduced the incidence of POUR (17). Lee and colleagues declared that POUR increases with age, with the risk increasing by 2.4 to 2.8 times in patients over 50 years of age (18). Although some studies have reported higher incidence of POUR in men compared with women, some studies have reported that there isn’t significant difference between men and women (3, 19). In our study, only men were included due to type of surgeries and limited number of female patients. The other limitation of our study was that we did not record perioperative fluid intakes of the patient’s.

There are various methods for prevention of POUR, such as induction of local instead of regional or general anesthesia, restriction of preoperative fluid intake, use of short acting anesthesia agent, early ambulation of patient’s after surgery, use of warm compress in suprapubic area and use of parasympathomimetic or α-adrenergic blockers (3, 4). In a review article published in 2010 to investigate the most effective drug for the treatment of POUR in adults, the authors concluded that no statistically significant associations were reported between successful treatment or any other outcome and alpha-blockers, cholinergic agents and sedatives as monotherapies. A statistically significant association between intravesically administered prostaglandin and successful voiding was detected. A statistically significant association was detected between cholinergic agents combined with sedative and an improved likelihood of spontaneous voiding compared with placebo (20).

The purpose of pharmacologic prevention of POUR is the increase of detrusor contractility or bladder neck and proximal urethral relaxation. Alpha-adrenergic receptors are found in trigone, prostatic urethra and ureters. These receptors cause contraction of the smooth muscles in these regions (21). Alpha-adrenergic blockers decrease bladder outlet resistance and facilitate micturation. Several studies found that prophylactic administration of alpha-blockers such as phenoxybenzamine and prazosin significantly decreases the incidence of POUR (10). Although all alpha-blocking compounds show similar levels of efficacy for lower urinary tract symptoms treatment, third generation alpha-blockers such as alfuzosin and tamsulosin tend to demonstrate improved selectivity for the prostate and bladder (22). Another advantage of tamsulosin and alfuzosin in the management of acute urinary retention is that a therapeutic dose can be administered at the onset of acute urinary retention (23). The mean time to peak serum concentration (Tmax) of alfuzosin and tamsulosin are 8 hours and 4-5 hours after an oral dose, respectively. Alfuzosin and tamsulosin have a serum half-life (T_1/2_) of 5 hours and 14-15 hours after oral administration, respectively (24). Madani et al. assessed preventive effect of tamsulosin on POUR after spinal anesthesia. In this randomized study, 118 patients received 0.4mg tamsulosin 14 and 2 hours before and 10 hours after surgery and 114 patients received placebo. They concluded perioperative administration of tamsulosin reduced the risk of POUR from 21.1% to 5.9% (10). In our study, tamsulosin 0.4mg were given orally 14 and 2 hours before surgery and alfuzosin 10mg were given orally 10 and 2 hours before surgery. The effectiveness of both of them on POUR had equal degree.

In the present study, 15 of 60 patients (25%) in the placebo Group had urinary retention. 3 of 60 patients (5%) in the tamsulosin Group and 4 of 60 patients (6.7%) in the alfuzosin Group had urinary retention and required catheterization. The incidence of POUR was significantly greater in men who did not receive tamsulosin or alfuzosin before surgery. The beneficial effects of tamsulosin and alfuzosin on POUR were similar.

## CONCLUSIONS

This study suggests that preoperative tamsulosin or alfuzosin administration reduces the incidence of postoperative urinary retention and the need for catheterization after surgeries under spinal anaesthesia. Therefore, the use of preoperative tamsulosin or alfuzosin can be recommended in adult male patients who will undergo urologic surgery under spinal anaesthesia.
